# Simulating arsenic migration in arid farmland soils under high-arsenic groundwater irrigation in Xinjiang

**DOI:** 10.1371/journal.pone.0351925

**Published:** 2026-06-24

**Authors:** Jiale He, Wenwen Deng, Tuerxun Tuerhong, Yanli Luo, Meijuan Wang, Zhen Song, Xuan Liu, Xinzhe Xie, Qian Zhang, Enmeng Yu, Rui Sun

**Affiliations:** 1 College of Resources and Environment, Xinjiang Agricultural University, Urumqi, Xinjiang Uygur Autonomous Region, China; 2 Institute of Agricultural Economics and Information, Xinjiang Academy of Agricultural Sciences, Urumqi, Xinjiang Uygur Autonomous Region, China; University of Peshawar National Centre of Excellence in Geology, PAKISTAN

## Abstract

Arsenic (As) is a toxic element widely distributed in the environment. A simulation irrigation experiment was conducted to investigate the migration characteristics of As(V) in farmland soils of the Kuitun River Basin, Xinjiang, under different irrigation scenarios. Irrigation water quality was simulated by altering evaporation intensities, pH values and TDS. Changes in As content in the soil solution were then analyzed. Ion exchange membranes were inserted directly into the soil to visualize As distribution in the soil profile, and the As adsorbed on the membranes was analyzed via SEM-EDS, providing spatial distribution information that corroborated the quantitative measurements of As in soil solution, thereby achieving visualization of As distribution within the soil profile. The results indicated that all three factors significantly influenced the distribution of As(V) in the top 0–10 cm of the soil layer. Under weak evaporation conditions, As migrated further both vertically and horizontally in the soil profile. Conversely, increasing the pH and salt content of the irrigation water promoted the downward penetration of As from the topsoil. These findings suggest that in the arid agricultural region, irrigation with less saline and alkaline groundwater may immobilize As effectively in the top soil layers and mitigate the potential risks associated with As soil contamination.

## 1. Introduction

In many regions, irrigation with high-arsenic (As) groundwater leads to arsenic accumulation in the surface soils of farmlands [1–8]. Under natural conditions, the As content in soil is typically low. However, the input of As into farmland soil due to human activities far exceeds the background value under natural conditions and leads to an increase in the content of As in agricultural soil at the local, regional, and even global levels [9–12]. Under natural conditions, the background concentration of As in soil typically ranges from 6–7 mg·kg^-1^; however, the repeated use of arsenic-contaminated groundwater for irrigation can potentially increase As concentrations above 10 mg·kg^-1^ [13]. This accumulation in farmland soils not only poses a threat to food security by reducing crop productivity but also poses health risks for individuals in direct contact and can eventually be transmitted to humans through the food chain [13]. The trend of As pollution in agricultural soils has gradually increased over the past three decades, and the geoaccumulation index revealed that arsenic in Chinese agricultural soil poses a low ecological risk to the ecosystem [14].

The migration behavior of As in soil varies significantly depending on the specific soil environment. Hao indicated that an increase in the Na^+^ concentration in a soil solution enhances the mobility of As(V) [15]. Nagar’s findings suggested that when the pH of a soil solution exceeds 7, the adsorption of As(V) in the soil decreases significantly, thereby affecting arsenic migration [16]. Zhong’s study demonstrated that reducing the pH and increasing the redox potential (Eh) can accelerate the release of arsenic in rice paddy soil [17]. Furthermore, the dissolution of As under oxidizing conditions and its desorption under reducing conditions are potential factors contributing to the mobilization of As in arid regions [18].

Xinjiang is a typical arid region characterized by the distribution of high-As groundwater. Specifically, these primary high-As groundwater resources are mainly concentrated in the Kuitun River Basin on the southern margins of the Junggar Basin [18–21]. This region serves as the largest oasis agricultural area in Xinjiang and ranks as the fourth largest irrigated agricultural region in China [22]. Given the limited availability of surface water resources in the region, groundwater has become the primary source for drinking, agricultural production, and domestic water usage [23]. Irrigation with high-As groundwater may lead to As migration in soils. For instance, Asamoah-Ntow et al. found in Ghana that As contamination in irrigation water leads to As accumulation in soils and poses risks to crop safety, indicating that high-As groundwater irrigation is a common challenge faced by arid and semi-arid regions [24]. Therefore, it is urgent to elucidate the migration and distribution characteristics of As under these soil conditions to assess the risk of As irrigation.

To investigate the migration patterns of As in soils, researchers have developed various methods. Field trials can provide direct evidence of As migration under natural conditions, but they are often limited by spatial heterogeneity and uncontrollable environmental factors [25–26]. Column leaching experiments conducted in the laboratory simulate the vertical migration of As in soils and can be used to study As transport under different irrigation regimes; however, they are constrained by in situ sampling limitations and cannot provide detailed information on As distribution across different soil layers [27]. In recent years, an innovative method combining ion exchange membranes with SEM-EDS technology has enabled the visualization of As distribution within soil profiles, offering spatial information that traditional methods cannot obtain [28]. This study aims to investigate As migration in soils under drip irrigation conditions. A simulated drip irrigation experiment combined with ion exchange membranes was employed.

In arid and semiarid regions, drip irrigation is a widely adopted water-saving technique. During the drip irrigation process, water undergoes spatial diffusion in the soil, ultimately forming a hemispherical wetting body. This is a characteristic moisture distribution pattern, where water accumulates near the drip emitter and decreases toward the edges. This unique distribution pattern can facilitate the leaching and redistribution of heavy metals from contaminated soil [29–30]. However, few studies have analyzed the specific distribution of As in soil after irrigation with As-contaminated water. To assess the risk of further migration of As within soil after its introduction more accurately, a more intuitive illustration of As distribution in soil following irrigation with As-laden water is necessary.

When designing controlled experiments of this nature, it is essential to recognize the complexity of As migration. Under natural conditions, the migration behavior of As in soils is influenced by a complex interplay of physical, chemical and biological factors. Physically, soil texture, pore structure and water flow pathways determine the transport of water-soluble As in the soil [31]. Chemically, pH, Eh, competing ions and organic matter collectively regulate the adsorption-desorption processes of As [26]. Biologically, soil microorganisms and plant roots can alter As speciation and the rhizosphere environment through metabolic activities or root exudates [27]. Among these factors, evaporation conditions, irrigation water pH and TDS are key environmental factors affecting As migration in arid soils. They not only directly influence the adsorption-desorption equilibrium of As in the soil but also indirectly modify As speciation and transport pathways by regulating soil water movement and ionic strength [32].

Based on this, a simulated irrigation controlled experiment was conducted to investigate the migration characteristics of As(V) in soils under different evaporation intensities, pH values and TDS conditions, using cotton field soils from the Kuitun River Basin in Xinjiang as the research object. The study employed an ion exchange membrane combined with SEM-EDS visualization method. Ion exchange membranes were inserted directly into the experimental soil to obtain two-dimensional distribution information of As within the soil profile, which was then corroborated with quantitative measurements of As in soil solutions. Compared with traditional stratified sampling, this method not only enables the determination of total As content in the soil but also intuitively reveals the vertical and horizontal migration patterns of As in the soil. The research findings are expected to provide a scientific basis for assessing the environmental risks of long-term high-As groundwater irrigation in arid agricultural regions.

## 2. Materials and methods

### 2.1. Study site

**Fig 1:**
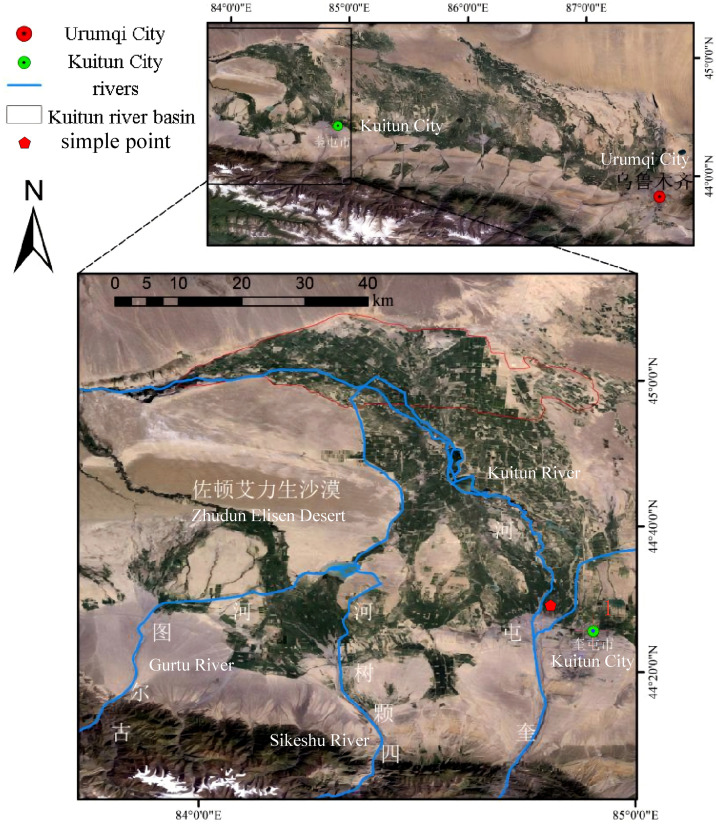
Map of the Kuitun river basin. Simple point is depicted as red pentagon.

This study was conducted in the Kuitun River Basin in northwestern China, as shown in Fig 1. The average pH of the groundwater used for farmland irrigation is 8.5, and the average TDS (total dissolved salts) is approximately 1.5 g·L^-1^ (Ca^2+^ 90 mg·L^-1^; Mg^2+^ 30 mg·L^-1^; K^+^ 0.12 mg·L^-1^; Na^+^ 390 mg·L^-1^; HCO_3_^–^60 mg·L^-1^; SO_4_^2–^540 mg·L^-1^; Cl^-^ 390 mg·L^-1^). These groundwater quality data were obtained from our research group’s previous study [33]. The tested soil was collected from the surface layer (0--30 cm) of a cotton field in the Kuitun River Basin. Soil samples were randomly collected from local farmland using a five-point sampling method. Prior to collection, surface debris and plant residues were removed. The subsamples from each plot were thoroughly mixed to form a composite sample, which was then air-dried, passed through a 5 mm sieve, and analyzed for its basic physicochemical properties, with the remaining soil reserved for subsequent experiments.

This study was conducted on privately owned cotton fields. Access to the fields and permission to conduct the research were granted orally by the individual landowners prior to the commencement of the study. No specific institutional or national permits were required for accessing these field sites, as the study did not involve entry to protected areas or interference with endangered species.

### 2.2. Experimental design

To investigate the migration characteristics of As(V) in farmland soil under different evaporation and irrigation conditions, eight treatments involving irrigation with As-containing saline water were designed, as shown in Table 1, and the main components of saltwater are shown in Table 2. The pH and TDS values for the different treatment groups were set based on the natural variation range of groundwater in the Kuitun River Basin to simulate both typical and extreme local irrigation water conditions. To ensure the accuracy of the experimental results, each treatment was performed in triplicate. The irrigation water was prepared using Na_3_AsO_4_·12H_2_O. Indoor treatment was conducted at 22 ~ 25°C (constant temperature conditions to minimize evaporation interference), whereas outdoor groups were subjected to outdoor treatment at 31 ~ 36°C (representing typical summer daytime temperatures in the region). The concentration of As(V) in the simulated irrigation water was 10.0 mg·L^-1^, approximately 10 times the maximum As(V) concentration (1.15 mg·L^-1^,) in the local irrigation water.

**Table 1:** Experimental design Note: CK represents the control group; E1 and E2 represent the indoor and outdoor treatment groups, respectively; P1, P2 and P3 represent the three pH treatment groups, respectively; T1, T2, and T3 represent the three TDS treatment groups, respectively.

Treatment	CK	E1	E2	P1	P2	P3	T1	T2	T3
pH	7.0	8.5	8.5	7.5	8.5	9.5	7.0	7.0	7.0
Evaporation condition	outdoor	indoor	outdoor	outdoor	outdoor	outdoor	outdoor	outdoor	outdoor
TDS (g·L^-1^)	0	1.5	1.5	0	0	0	0.5	1.5	4.5
As (g·L^-1^)	0	10.0	10.0	10.0	10.0	10.0	10.0	10.0	10.0

**Table 2:** Irrigation water salinity setting (mg·L^-1^,).

Total salt	Ca^2+^	Mg^2+^	K^+^	Na^+^	HCO_3_^-^	SO_4_^2-^	Cl^-^
500	30	10	0.04	130	20	180	130
1500	90	30	0.12	390	60	540	390
4500	270	90	0.36	1170	180	1620	1170

**Fig 2:**
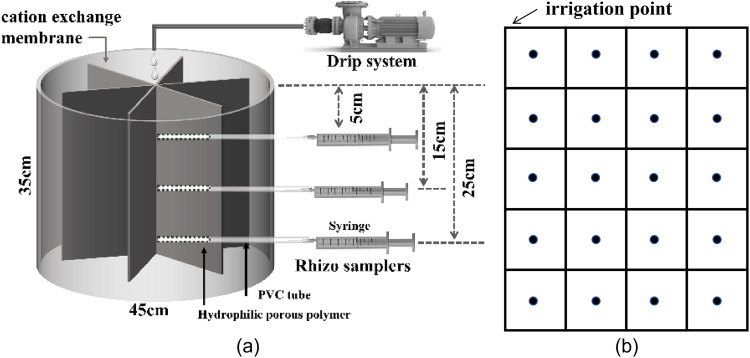
Schematic diagram of dripping irrigation, soil water sampling and the position of ion exchange membrane. **(a)** Schematic diagram of the experimental setup **(b)** Measurement points of As on the ion exchange membrane.

The soils (20 kg) were packed into cylindrical pots with a diameter of 45 cm and a height of 35 cm (dry bulk density of 1.3 g/cm^3^), as shown in Fig 2(a). To visualize the distribution of As content in the soil profile after the simulated irrigation, three cation exchange membranes with a size of 20 cm × 30 cm (produced by Hangzhou Lanran Technology Company) were vertically inserted into the soil of each pot [34]. To simultaneously capture the vertical and horizontal migration characteristics of As within the soil profile, the three membranes were cut in the middle and inserted into the soil at a 60° angle, allowing them to intercept As diffusing from the irrigation point. An automatic drip irrigation system dripped water containing 10 mg·L^-1^ As(V) into the center of the pot at a rate of 5 mL·min^-1^ for 200 minutes per day in the evening to eliminate the effect of daytime evaporation (except for E1). Drip irrigation was continued for 10 days. Soil solution sampling tubes (Rhizon MOM, sampling head length 10 cm, 2.5 mm OD, Rhizophere Research Products, Netherlands) with filter tube membranes with a pore size of 0.60 μm were embedded into the soil at depths of 5 cm, 15 cm and 25 cm, as shown in Fig 2(a). The positions of the samplers were fixed during the experiment. The soil solutions were collected continually by sampling tubes and transferred into centrifuge tubes for total As determination. After the experiment (21 days), the membranes were collected from the soil for analysis.

### 2.3. Measurement methodologies

Soil particle size distribution was determined using a laser particle size analyzer (Mastersizer 3000, Malvern, UK). Soil pH was measured with a pH meter (FE28-Meter). Oxidation-reduction potential (Eh) was determined using an oxidation-reduction potentiometer (QX6530). Electrical conductivity (EC) was measured with a conductivity meter (DDS-307). Soil organic carbon was determined by the potassium dichromate oxidation-spectrophotometry method. The contents of Mn and Fe in the soil were determined by flame atomic absorption spectrophotometry (TAS-990) after digestion with HNO_3_-HClO_4_. Soil As content was determined by atomic fluorescence spectrometry (PF-3) after digestion with HNO_3_-HClO_4_. All measurements were performed in triplicate, and the results were expressed as mean values.

The Eh, pH and total As in the soil solution were measured via a DZB-718-B portable multiparameter water quality analyzer, a portable precision TDS meter and a PF-3 atomic fluorescence spectrometer (HJ 694-2014) after HNO_3_-HClO_4_ digestion, respectively. The measured values were subtracted from the values in the CK treatment.

Prior to determining the distribution of As on the ion exchange membrane, the soil particles that adhered to the surface of the membrane were removed thoroughly via a small brush to avoid the effects of As in the soil particles on the results of SEM scanning. Once cleaned, the membrane was cut into 5 cm × 5 cm pieces as shown in Fig 2(b), and the distribution of As on each piece of membrane was determined via SEM‒EDS (SEM 500 field emission scanning electron microscope; Aztec X-Max 50 energy-dispersive X-ray spectroscopy). The scanning electron microscope operating conditions were set at an accelerating voltage of 10 kV, a working distance of 10 mm, and an energy spectrum collection time of approximately 2 minutes per point.

### 2.4. Data analysis

All the tests were performed in triplicate, and the experimental data were statistically analyzed with Excel 2019. One-way analysis of variance (ANOVA) was used to test the significance of differences in As content among different treatments, with *P* < 0.05 considered statistically significant. The contour map of the As content on the ion exchange membrane was plotted by kriging interpolation via the software Surfer 13.0 using default parameter settings. The migration characteristics of As under different treatments were evaluated by analyzing the apparent As content at different positions on the membranes in conjunction with the measured As data from soil solutions at different depths.

### 2.5. Quality control

To ensure the accuracy and reliability of the analytical results, strict quality control measures were implemented during the experiment. Three blank samples were set in each batch of determinations to monitor potential contamination during the experimental process, and the measured values of the blank samples were all below the method detection limit. The atomic fluorescence spectrometer was calibrated with standard solutions before each use, and the correlation coefficients (R^2^) of the calibration curves were all greater than 0.999.

The detailed EDS scan results and soil solution data under different pH, TDS, and evaporation conditions are provided in S1-S4 Files.

## 3. Results

### 3.1. Basic physicochemical properties of the tested soil

The basic physicochemical properties of the tested soil are shown in Table 3. The soil particle size distribution consisted of 28.67% sand (2 ~ 0.02 mm), 36.29% silt (0.02 ~ 0.002 mm), and 35.04% clay (<0.002 mm), with a loamy clay texture. The soil pH was 7.86, indicating weak alkalinity; the electrical conductivity was 2.93 mS·cm^-1^, suggesting a certain degree of salt accumulation. The soil organic carbon content was 3.47 g·kg^-1^, which is at a relatively low level among farmland soils in arid regions of Xinjiang. The background contents of As, Mn and Fe in the soil were 9.98, 0.53 and 13.99 mg·kg^-1^, respectively. The As content was close to the background range of farmland soils in Xinjiang, indicating that the tested soil was not significantly contaminated by exogenous As.

**Table 3:** Basic physicochemical properties of test soil soil soil chemical composition.

proportion of particles (%)			soil texture	Conductivity (mS·cm^-1^)	pH	total chemical composition (mg·kg^-1^)			organic carbon (g·kg^-1^)
sand (2 ~ 0.02 mm)	silt (0.02 ~ 0.002 mm)	clay (< 0.002 mm)				As	Mn	Fe	
28.67	36.29	35.04	loamy clay	2.93	7.86	9.98	0.53	13.99	3.47

### 3.2. Migration characteristics of As under different irrigation conditions

#### 3.2.1. Migration characteristics of As under different pH conditions.

**Fig 3:**
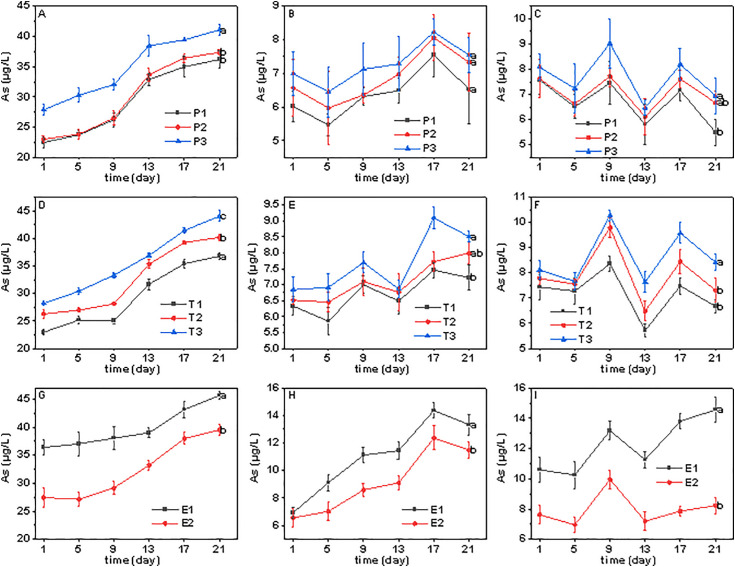
Time variation of As content in extracted soil solution in different treatments. In each row, from left to right, the figures represent the As content in the soil solution at depths of 5 cm, 15 cm and 25 cm for different treatment groups.Fig. A, B and C represent the penetration of As under varying pH conditions, correspond to treatment P1 (pH = 7.5), P2 (pH = 8.5) and P3 (pH = 9.5), respectively; Fig. D, E, and F represent the penetration of As under different TDS conditions, correspond to treatment T1 (TDS = 0.5g/L), T2 (TDS = 1.5g/L) and T3 (TDS = 4.5g/L), respectively; Fig. G, H, and I indicate the penetration of As under various evaporation conditions, correspond to treatment E1 (indoor) and E2 (outdoor), respectively; Different lower-case letters near the error bar indicate significant differences in As content between different depths (*P* < 0.05);.

Fig 3 shows the dynamic changes in the As content in the soil solutions at depths of 5 cm, 15 cm and 25 cm under different pH conditions. As depicted in Fig 3A, B and C, the As content in the soil solution at a depth of 5 cm for the three treatments increased steadily. P3 had the highest As content during all experimental periods and was significantly greater than P2 and P1 (P < 0.05) during the entire trial period, followed by P2 and P1, indicating that pH 9.5 hindered the adsorption of As onto the soil surface more than pH 8.5 and pH 7.5. There was no significant difference between P1 and P2 at the end of the experiment (*P* > 0.05). Under deeper soil conditions, no significant differences in the As content of the soil solution were detected among the different treatments at depths of 15 cm and 25 cm (*P* > 0.05). However, the As content at a soil depth of 15 cm tended to increase, whereas the As content at a depth of 25 cm tended to decrease in all three treatments.

#### 3.2.2. Migration characteristics of As under different salt conditions.

Fig 3, D, E and F show the changes in As content in the soil solutions at depths of 5 cm, 15 cm and 25 cm under different salt conditions. The As content in the solution collected from a depth of 5 cm tended to increase in all the treatments. The soil As increased with increasing water salt content, i.e., the soil irrigated with the saltiest water (T3) presented the highest As content at all soil depths, followed by those in T2 and T1. Multiple comparisons revealed significant differences (*P* < 0.05) in the soil As contents among the three treatments at a depth of 5 cm and between T3 and T2/T1 (*P* < 0.05) at a depth of 25 cm. In short, these observations suggest that a high salt content facilitates the accumulation of free As ions in soil water.

#### 3.2.3. Migration characteristics of As under different evaporation conditions.

Fig 3, G, H, I. Effects of indoor and outdoor conditions on the changes in As in soil solutions at depths of 5 cm, 15 cm and 25 cm. The As content in the soil solutions of both E1 (indoor group) and E2 (outdoor group) increased gradually over time. The As contents in E1 at all depths were greater than those in E2, and statistical analysis revealed a significant difference in soil As between E1 and E2 at all soil depths (*P* < 0.05). The differences between E1 and E2 were more significant in deeper soil. This result indicated that indoor conditions with less evaporation led to more free As ions in arid farmland soil.

### 3.3. As distribution in soil

**Fig 4:**
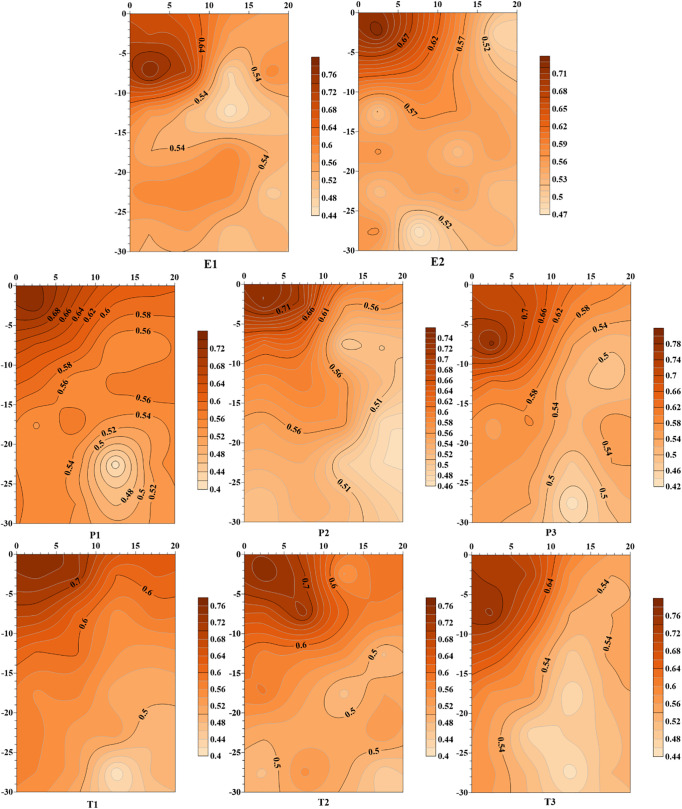
Contour map of As content on the ion exchange membranes in different treatments. The value depicted in the figure represents the relative contents of As generated by SEM-EDS scanning.

Fig 4 shows the contour maps of the As concentration on the ion exchange membrane. The color depth and corresponding digital values represent the intensity of signals generated from the As atoms on the surface of the membrane, indicating that the higher the values are, the higher the As concentration. In the E1 group, the As values ranged from 0.44 to 0.78, with the maximum value occurring at a depth of 8 cm and at a horizontal distance of 3 cm from the center of the pot. In contrast, the E2 group presented an As value ranging from 0.47 to 0.73, with the highest As value observed at a depth of 2 cm and a horizontal distance of 2.5 cm from the pot center. This result revealed that the As in the case of E1 migrated vertically deeper than those in the E2 group did, indicating strong vertical downward penetration of free As ions under weak evaporation conditions. This finding is consistent with the results in Section 3.2.3, where the E1 treatment exhibited significantly higher As concentrations in the soil solution at all depths compared to E2, confirming that under weak evaporation conditions, upward water movement is reduced while downward leaching is enhanced, leading to the migration of As to deeper soil layers.

In the P1, P2 and P3 treatment groups, the As values ranged from 0.4 to 0.74 in the P1 group. The highest concentration was observed at a depth of 3 cm and a distance of 4 cm from the center of the pot. Similarly, in the P2 group, As ranged from 0.46 to 0.74, with the maximum concentration occurring at the same location as in P1. In contrast, the P3 group presented an As range of 0.42 to 0.80, with the highest concentration recorded at a depth of 7.5 cm and a distance of 2.5 cm from the pot center. The As in P3 migrated much deeper than those in P1 and P2 did, suggesting that the higher pH of the irrigation water facilitates the transport of As ions to deeper soil layers. This result is consistent with the finding in Section 3.2.1 that the P3 treatment had the highest As content in the soil solution at a depth of 5 cm, indicating that high pH not only promotes the desorption of As from the soil surface but also enhances its migration to deeper soil layers.

In the T1, T2 and T3 treatment groups, As ranged from 0.4 to 0.76 in the T1 group, and the highest concentration was observed near the drip point of the irrigation water. For the T2 group, As varied from 0.44 to 0.76, with two peaks at depths of 3 cm and 4 cm close to the drip point of the irrigation water. In the T3 group, the As values were between 0.44 and 0.78, and the highest concentration region was found at a depth of 8 cm and a distance of 2 cm from the center of the pot. Combined with the results in Section 3.2.2, where the T3 treatment exhibited the highest As content in the soil solution at all depths, these findings indicate that high salinity in irrigation water increases the content of free As ions in the soil solution and accelerates the vertical leaching of As. In short, As migrated deeper in the T3 group than in the T1 and T2 groups, suggesting that the salinity of the irrigation water facilitates the vertical migration speed of As ions in the soil.

## 4. Discussion

Soil water is the most dynamic component in soils and serves as the venue for the migration of water-soluble components and chemical reactions [35]. Our experimental observations revealed that As introduced into the soil through irrigation migrates slowly and mainly accumulates in the topsoil layer, ranging from 0--10 cm. Deng et al. noted that the soil in this region has a high affinity for adsorbing As(V) and a low tendency for desorption, indicating that As is rapidly immobilized within the soil upon entry through irrigation water, thus hindering its downward migration [36]. This phenomenon is mainly related to the mineral composition of the local soil, which is rich in Fe and Mn oxides. These minerals possess a large number of variable charge sites on their surfaces and exhibit strong specific adsorption for As(V) through coordination, forming stable inner-sphere complexes with As, thereby inhibiting As migration [37]. Okonkwo et al. reported that the total As concentration in contaminated soil decreases with depth and peaks at 0 cm [38]. Similarly, Frentiu et al. studied the distribution of As(V) in hand-dug wells in Baia Mare, Romania, and reported that the migration rate of As(V) decreases with increasing soil depth [39]. These findings are consistent with our experimental results, indicating that the As introduced into the soil through irrigation is primarily concentrated within the 0--10 cm depth range.

Further investigation using ion exchange membranes combined with SEM-EDS revealed that, under outdoor irrigation with high-As solution (pH = 8.5, TDS = 1.5 g·L^-1^,), As migration occurred within 2 cm in both vertical and horizontal directions. Traditional leaching experiments can only quantify the total As content in soil solutions at different depths, but cannot distinguish the spatial distribution characteristics of As within the soil profile. This is consistent with the findings of Li et al., who studied As migration in soils of the Kuitun River Basin using leaching experiments and reported that As was mainly concentrated within the 0 ~ 2 cm soil layer [40]. In this study, the ion exchange membrane combined with SEM-EDS technique intuitively revealed that As does not migrate uniformly downward; instead, it forms a “hemispherical” high-As zone near the drip emitter. The soil has a strong adsorption capacity for As, which tends to be immobilized upon entering the soil, resulting in limited downward migration with the solution.

The environmental behavior of As in soil is influenced by various environmental factors, among which pH and Eh are particularly crucial. Changes in soil pH directly impact the adsorption and desorption processes of As, whereas an increase in Eh can promote the formation of mobile As species and competitive ions for As [41–42]. Under alkaline conditions, the degree of deprotonation on soil mineral surfaces increases, leading to more negative surface charges and weakened electrostatic adsorption of As(V) anions [43–47]. Conversely, a lower pH favors the migration of As from the upper soil layers to lower depths [48]. Studies have shown that in addition to promoting the transformation of soil As into water-soluble forms and increasing its migration risk in the soil environment, higher pH also facilitates the conversion of As into other bioavailable species [47,49]. Furthermore, high pH soil environments can directly disrupt the stability and function of plant root cell membranes, altering their normal absorption and transport of water and nutrients. Under the combined influence of high pH and elevated As content in the environment, plant tolerance to As toxicity may decrease, subsequently exacerbating the inhibitory effects of As exposure on crop growth [49].

The salinity of irrigation water also significantly affects the distribution and migration of As in soil. Higher As concentrations in soils are associated with high salt contents [50–51]. In our experiments, the increasing trend of the As content in the soil solution with increasing irrigation water salinity suggested that soil-bound As was released from the soil surface through the influence of higher ion concentrations. Under high salinity conditions, the increased concentration of cations in the soil solution compresses the electrical double layer on the soil mineral surfaces, reducing the electrostatic adsorption between soil minerals and As(V) [52]. Simultaneously, anions such as Cl^-^ and SO_4_^2-^ can compete with As(V) for adsorption sites, displacing As through ion exchange. These findings were consistent with the results of the previous studies mentioned above.

In addition to irrigation water conditions, evaporation is also a significant factor influencing the migration and distribution of As in soil. In such systems, dissolved As in the soil accumulates due to evaporation from the surface and aeration zones [5]. As in irrigation, water migrates along with the movement of water once it enters the soil [53]. Under strong evaporation conditions, upward water movement dominates, forming an upward hydraulic gradient that carries dissolved As to the surface for accumulation [54]. Under weak evaporation conditions, gravity-driven downward percolation dominates, and As leaches downward with the water [55]. Therefore, under strong evaporation, the upward migration of water in the soil carries the desorbed As, which might explain the greater migration distance of As observed in indoor environments with weak evaporation.

Combining our findings with the results of similar studies mentioned above, it can be deduced that the higher pH and salt content of irrigation water hinder the adsorption of As onto the soil surface and promote the release of soil-bound As. In the study area of this region, it is imperative to fully consider local irrigation conditions and soil properties to adopt appropriate measures for reducing the accumulation of exogenous As in the soil and the release of soil-bound As. By reducing the soil pH and salt content, the potential risks associated with As contamination in agricultural soil can be effectively mitigated, and the safety of agricultural products and the sustainability of agricultural ecosystems can be safeguarded.

## 5. Conclusions

Evaporation and irrigation with As-containing saline‒alkali water significantly influenced the migration of As on the soil surface in the Kuitun River Basin of Xinjiang. With respect to the soil depth, As accumulated more over time in the topsoil after being irrigated with saline water containing 10 mg kg^-1^ As, whereas the accumulation of As in deeper soil decreased with increasing time. The salt content and pH of the irrigation water and evaporation intensity did not affect the accumulation of As in the topsoil. However, the relatively high salt content and pH of the irrigation water significantly increased the As concentration in the topsoil. The As concentration in the soil solution at all soil depths was greater under weak evaporation intensity conditions. These factors also influenced the distribution of As in the topsoil. In the case of intense evaporation, the migration depth and distances of As (in the vertical and horizontal directions) within the topsoil are significantly short. Elevating the pH and salt content of irrigation water increased the vertical downward migration of As once it entered the soil. It can be inferred that using low-salinity and weakly alkaline irrigation water in this study area may reduce the environmental risk of As (V) in the soil.

## Supporting information

S1 File Soil solution data at different pH levels. (XLSX)

S2 File Soil solution data at different TDS levels. (XLSX)

S3 File Soil solution data under varying evaporation conditions. (XLSX)

S4 File EDS scan results. (XLSX)
