# Supplementary material for: Simulating arsenic migration in arid farmland soils under high-arsenic groundwater irrigation in Xinjiang
Source: PLoS One. 2026 Jun 24;21(6):e0351925. doi: 10.1371/journal.pone.0351925 (PMC13293429; doi:10.1371/journal.pone.0351925)
Supplement: Attachment — Submitted filename: Response to Reviewers.docx [file pone.0351925.s006.docx]

**Response to the Reviewer’s Comments**

**Manuscript number:** PONE-D-25-46672

**Title:** The migration of arsenic in the arid farmland soil under arsenic groundwater irrigation in Xinjiang

Dear Editors and Reviewers,

We appreciate the opportunity to revise our manuscript titled *The migration of arsenic groundwater irrigation in Xinjiang* and are grateful for the insightful comments provided by the reviewers. Those comments are all valuable and very helpful for revising and improving our paper, as well as the important guiding significance to our researches. In the following, we have provided detailed responses to each of the reviewers' comments. Revised portion are marked in red in the paper. Additionally, we have conducted a comprehensive revision of the entire manuscript. In this response letter, the reviewers' comments are presented in italics. We have tried our best to make all the revisions clear, and we hope that the revised manuscript meets the requirements for publication.

------------------------------

Response to Reviewer #1

**Reviewer Comments 1：** *Figure captions need to be explanatory like P1，P2, and P3 which pH ranges each one is referring same is the case with other treatments.*

**Response :** Thank you for this valuable suggestion. We have revised the captions for all figures to clearly indicate the specific conditions for each treatment. In Fig 1, we have added a note explaining that P1, P2 and P3 correspond to pH 7.5, 8.5 and 9.5, respectively, and that T1, T2 and T3 correspond to TDS 0.5, 1.5 and 4.5 g·L^-1^, E1, E2 correspond to indoor and outdoor treatment, respectively. The revised can be found in the document titled Figures in red.

**Reviewer Comments 2：** *pH 9.5 although hindered Arsenic adsorption on the soil surface. What is the impact of this soil pH on plant growth?*

**Response :** Dear reviewer, thank you for your raising on this important point. In the revised Discussion section, we have added a paragraph addressing the impact of high pH on plant growth. Specifically, we discuss how high pH environments can disrupt root cell membrane stability and function, altering water and nutrient uptake, and how this, combined with elevated As bioavaliability, may exacerbate the inhibitory effects of As on crop growth. This addition can be found on Page 18, Lines 360-368.

**Reviewer Comments 3：** *Figure 2 Captions need to be more clearly explained. Figure 2 (A-C) No depth point has been mentioned in the figure panel or in the caption. How significant was the difference at which time point in case of P3. Figure 2 (D-F) Concentration of salt solution used for analysis not mentioned. Again the depth should be mentioned in the figure panel/caption.*

**Response :** Thank you for your suggestion. we have revised the figure captions accordingly. In response to Reviewer 4’s comments on adding a map, the original Fig 2 has changed to Fig 3. In the caption for Fig 3, we now explicitly state that the data points at depths of 5 cm, 15 cm and 25 cm are shown from left to right. For the P3 treatment, we have clarified that the As content was significantly higher than P1 and P2 throughout the entire experimental period, as stated on Page 12, Lines 235-238. The salt concentrations for treatments T1, T2 and T3 (0.5, 1.5 and 4.5 g·L^-1^) are now clearly indicated in the figure captions and Page 8, Table 1.

------------------------------

Response to Reviewer #2

**Reviewer Comments 1 (Objective)：** *The objective of the study is not clearly stated. The objective and title must be described in accordance with the methodology employed and in agreement with the experimental results. This is particularly important because the study is based on controlled experimental conditions rather than direct field application; therefore, the innovative contribution of the present work should be explicitly stated. Furthermore, the abstract mentions that the study area is a reclamation area; however, this is not subsequently reflected in the objective proposed in the Introduction section. Additionally, this creates confusion in the Study Area section (Methodology).*

**Response :** Thank you for this important observation. We have changed the title to *Simulating arsenic migration in arid farmland soils under high-arsenic groundwater irrigation in Xinjiang* to highlight the simulation of the experiment and revised the objective statement in the Introduction to clearly reflect the controlled experimental nature of the study and to highlight the innovative contribution of our methodology. The revised objective now reads: "Based on this, a simulated irrigation controlled experiment was conducted to investigate the migration characteristics of As(V) in soils under different evaporation intensities, pH values and TDS conditions, using cotton field soils from the Kuitun River Basin in Xinjiang as the research object." This can be found on Page 5-6, Lines 103-106. We have also ensured consistency between the abstract and the Introduction by specifying "Kuitun River Basin" throughout the manuscript.

**Reviewer Comment 2 (Introduction):** *Although the Introduction mentions the arsenic situation in the area of interest, it should also describe the different methodologies available for assessing arsenic mobilization, as well as the physical, chemical, and biological dynamics that must be considered in this type of controlled experiment. This is relevant because the study focuses on evaluating the mobilization of this metalloid within a simulated environment.*

**Response:** We agree with the reviewer that a comprehensive methodological background is essential. In the revised Introduction, we have added a paragraph (Page 4, Lines 67-79) that reviews various methods for studying As migration, including field trials, column leaching experiments, and the innovative ion exchange membrane combined with SEM-EDS technique. We have also added a paragraph (Page 5, Lines 90-102) discussing the physical, chemical, and biological factors that influence As behavior in controlled experiments, and explaining why we focused on evaporation, pH, and TDS as key variables in this study.

**Reviewer Comment 3 (Study site):** *In this subsection, some results from soil characterization are reported. However, this section should be written in a way that clearly explains that this area corresponds to the source from which soil samples were collected. This can be addressed in different ways: a) This subsection could be removed, integrating the description of the soil sampling location into the experimental design. b) If this subsection is retained, the area should be described in general terms, including physical aspects such as location, hydrological, climatic, geological/edaphological characteristics, and socioeconomic activities, without repeating information already presented in the Introduction. c) Are the values of the physicochemical variables for both groundwater and cultivated soil characterization own analyses or obtained from literature? If they are own results, they should be reported in the Results section; if they are taken from previous studies, the corresponding bibliographic references must be provided.*

**Response:** Thank you for your three suggestions. Explaining the source of the samples can indeed facilitate readers’ understanding of our research, which is very important to us. We choose to combine suggestions a) and c) for modification. We have revised Section 2.1 to focus on general geographical and environmental descriptions of the Kuitun River Basin. We have clarified that the soil samples were collected from this area and that the groundwater quality data were obtained from our research group's previous study (Deng et al. 2021). The soil characterization results have been moved to the Results section (Section 3.1) as suggested. The revised text can be found on Page 6-7, Lines 122-129 and Page 11-12, Lines 223-236.

**Reviewer Comment 4 (Experimental design):** *The experimental design is not clearly described as currently presented. This subsection should comprehensively detail all procedures performed: sampling sites (number of soil and water sampling sites, and whether they were statistically determined), depth and area (sampling methodology), number of samples per site (quality control, whether composite samples were prepared), soil sample characterization procedures, number of water samples characterized, and whether the water used in the experiment was groundwater or a prepared solution. The basis for selecting pH and salinity values in the water should be clearly described. The meaning of CK, E, and P in Table 2 must be explained. The number of cylindrical pots used per treatment type (TDS, pH, and salinity) should be specified, as well as the rationale for selecting surface and indoor temperatures and the chosen TDS, pH, and salinity values. The criteria for using the membrane arrangement shown in Figure 1 should also be justified. All analyzed variables must be listed, as the Results section reports Eh measurements that are not mentioned in this section.*

**Response:** Thank you for patiently pointing out the shortcomings in our experimental design description. We have substantially expanded Section 2.2 to include all requested details:

Sampling method: Five-point sampling method, composite sample preparation (Page 6, Lines 124-126)

Water source: Laboratory-prepared solution using Na₃AsO₄·12H₂O (Page 7, Line 144-145)

pH and TDS selection: Based on natural variation range of groundwater in the Kuitun River Basin (Page 7, Lines 140-143)

Treatment abbreviations: Explained in Table 1 note (Page 8, Lines 152-154)

Number of replicates: Each treatment performed in triplicate (Page 7, Line 143-144)

Temperature rationale: Indoor 22–25°C (constant temperature to minimize evaporation), outdoor 31–36°C (typical summer daytime temperatures) (Page 7, Lines 145-148)

Membrane arrangement: Justified on Page 8-9, Lines 159-166 and supplemented with references on the use of similar ion membrane installation methods.

All analyzed variables: Eh, pH, total As in soil solution, and As distribution on membranes are now explicitly listed (Page 9-10, Lines 178-203)

**Reviewer Comment 5:** *A specific subsection describing the measurement methodologies should be included, covering elemental and physical determinations, as well as a detailed description of the SEM-EDX methodology (morphological and semi-quantitative analysis).*

**Response:** Thank you for your advice. We have added Section 2.3 "Measurement methodologies" (Page 9-10, Lines 178-203) that includes detailed descriptions of all analytical methods, including SEM-EDS operating conditions (accelerating voltage 10 kV, working distance 10 mm, acquisition time ~2 min per point).

**Reviewer Comment 6 (Data analysis):** *The statistical analyses used should be clearly specified and justified. Regarding the kriging evaluation applied to the membrane, it should be clarified whether arsenic concentration was assessed vertically, according to the arrangement shown in Figure 1. The characteristics and parameters of the kriging method applied must be described.*

**Response:** Dear reviewer, we have revised Section 2.4 to specify that one-way ANOVA was used to test significance (P < 0.05). For the kriging analysis, we clarified that vertical As migration was assessed by analyzing membrane sections at different depths, and horizontal migration by comparing sections at the same depth but different radial positions, and supplemented the content of Fig 2 to facilitate readers’ understanding of the method for determining arsenic content on ion exchange membranes. Default kriging parameters in Surfer 13.0 were used, as the focus was on visualizing relative distribution patterns. These details can be found on Page 10-11, Lines 204-213.

**Reviewer Comment 7 (Results and discussion):** *Because the experimental design is not clearly defined, the results lack a general framework that explains what is being analyzed to address the stated objective. The analysis of treatments is presented separately; however, the relationships among treatments and their connection with the results obtained from the membrane are not clearly established, and the analysis of the membrane itself is not well understood. To strengthen the manuscript, figures showing morphology and a table with qualitative elemental analysis derived from SEM-EDX should be included.*

**Response:** We have restructured the Results section to improve clarity and establish clear connections between the soil solution data and membrane results. Section 3.2 presents the soil solution data for pH, salt, and evaporation conditions, while Section 3.3 presents the membrane results, with each subsection explicitly referencing the corresponding findings from Section 3.2.(Page 14-16, Lines 279-320).

------------------------------

Response to Reviewer #3

**Reviewer Comments：** *While the topic of arsenic migration in arid agricultural soils is relevant, the manuscript suffers from fundamental scientific and methodological weaknesses that rule out publication in its current form. The experimental design is overly simplified and does not realistically represent arsenic geochemical behavior in soils, particularly with respect to redox control, mineralogical interactions, and arsenic speciation. The central reliance on ion-exchange membranes combined with SEM-EDS provides only semi-quantitative, unvalidated evidence, yet the results are interpreted as definitive spatial distributions. Data analysis is largely descriptive, lacks proper statistical or mechanistic treatment, and does not advance process-level understanding beyond well-established concepts.*

**Response：**We thank the reviewer for their critical assessment, which has helped us significantly improve the manuscript. We have addressed these concerns as follows:

1. Experimental design complexity: In the revised Introduction (Page 5, Lines 90-102), we now explicitly acknowledge the complexity of As migration in natural systems, including redox control, mineralogical interactions, and biological factors. We explain that our study focuses on three key factors (evaporation, pH, and TDS) as a controlled approach to isolate their effects, while recognizing that these interactions occur within a more complex natural system.
2. Methodology validation: We have clarified that the ion exchange membrane combined with SEM-EDS provides spatial distribution information that **corroborates** the quantitative measurements of As in soil solutions, rather than replacing them. This distinction is now clearly stated in the Abstract (Page 2, Lines 14-18) and in Introduction (Page 6, Lines 106-110). The two methods are presented as complementary rather than definitive alone.
3. Statistical and mechanistic analysis: We have strengthened the data analysis by:

·Adding one-way ANOVA to test significance of differences among treatments (Section 2.4, Page 10-11, Lines 206-208);

·Incorporating mechanistic explanations in the Discussion, including mineral adsorption mechanisms (Fe/Mn oxides forming inner-sphere complexes), pH effects (OH^-^ competition, electrostatic adsorption), salinity effects (double layer compression, ion exchange), and evaporation effects (hydraulic gradients). These can be found throughout Section 4 (Page 16-18, Lines 328-333, 357-359, 374-378, 384-387).

4. Process-level understanding: The revised Discussion now advances beyond well-established concepts by:

·Revealing the "hemispherical" high-As zone near the drip emitter, which traditional methods cannot capture

·Demonstrating that high pH and salinity not only increase As release but also change its migration direction from horizontal diffusion to vertical leaching

·Providing spatial evidence that distinguishes between desorption and migration processes

We believe these revisions have substantially strengthened the scientific rigor of our study and clarified its contribution to understanding As migration under controlled irrigation conditions.

------------------------------

Response to Reviewer #4

**Reviewer Comments 1：** *Abstract should be revised, objective clearly stated and also methodology the conclusion should be based on the results. Sentences are too long (e.g., line 13 to 16).*

**Response:** Thank you for your suggestions. We have revised the Abstract to ensure the objective is clearly stated, the methodology is concise, and the conclusions are directly based on the results. Long sentences have been split into shorter, clearer statements. The revised Abstract can be found on Page 2, Lines 9-25.

**Reviewer Comment 2:** *Introduction - add current references, some references are too old. Strengthen the problem statement.*

**Response:** Thank you for your advice, adding recent references can facilitate readers to have a clearer understanding of the current research status. We have added several recent references (2020-2025) throughout the Introduction, including Asamoah-Ntow et al. (2025), Tran et al. (2023), Viana et al. (2022), and Morosini et al. (2023). The problem statement has been strengthened by adding a global perspective: "For instance, Asamoah-Ntow et al. found in Ghana that As contamination in irrigation water leads to As accumulation in soils and poses risks to crop safety, indicating that high-As groundwater irrigation is a common challenge faced by arid and semi-arid regions" (Page 4, Lines 62-65).

**Reviewer Comment 3:** *Methodology - add map of study area.*

**Response:** A map of the study area has been added as Fig. 1, showing the location of the Kuitun River Basin in Xinjiang, China.

**Reviewer Comment 4:** *Data analysis - indicate the version of Excel.*

**Response:** We have specified that Excel 2019 was used for statistical analysis (Page 10, Line 205-2206).

**Reviewer Comment 5:** *Add method validation/control for the instrument etc.*

**Response:** We have added Section 2.5 "Quality control" (Page 11, Lines 214-221) describing the use of blank samples, calibration standards, and acceptance criteria (R² > 0.999).

**Reviewer Comment 6:** *Discussion should be strengthened.*

**Response:** The Discussion has been substantially strengthened with:

·Mechanistic explanations for all observed phenomena

·Comparison with traditional methods to highlight innovation

·Integration of soil solution data with membrane results

·A comprehensive summary of the study’s contributions

These revisions can be found throughout Section 4 (Page 16-19, Lines 321-399)

------------------------------

We hope that the revised manuscript now meets the standard for publication in PLOS ONE. We are grateful for the reviewers’ constructive comments, which have significantly improved the quality of our work.

Thank you for your time and consideration.

Sincerely,

Yanli Luo

Corresponding author

College of Resources and Environment

Xinjiang Agricultural University

Email: Luoyl@xjau.edu.cn
