# Supplementary material for: Simulating arsenic migration in arid farmland soils under high-arsenic groundwater irrigation in Xinjiang
Source: PLoS One. 2026 Jun 24;21(6):e0351925. doi: 10.1371/journal.pone.0351925 (PMC13293429; doi:10.1371/journal.pone.0351925)
Supplement: Peer review history

# Peer review: Decision Letter 0

**DOI:** 10.1371/journal.pone.0351925.r001

**Authors:** Wajid Ali

17 Feb 2026

PONE-D-25-46672

The migration of arsenic in the arid farmland soil under arsenic groundwater irrigation in Xinjiang

PLOS One

Dear Dr. Luo,

Thank you for submitting your manuscript to PLOS ONE. After careful consideration, we feel that it has merit but does not fully meet PLOS ONE’s publication criteria as it currently stands. Therefore, we invite you to submit a revised version of the manuscript that addresses the points raised during the review process.

Comments by the reviewers are uppended below.

Please submit your revised manuscript by Apr 03 2026 11:59PM. If you will need more time than this to complete your revisions, please reply to this message or contact the journal office at plosone@plos.org. When you're ready to submit your revision, log on to https://www.editorialmanager.com/pone/ and select the 'Submissions Needing Revision' folder to locate your manuscript file.

Please include the following items when submitting your revised manuscript:

- A letter that responds to each point raised by the academic editor and reviewer(s). You should upload this letter as a separate file labeled 'Response to Reviewers'.
- A marked-up copy of your manuscript that highlights changes made to the original version. You should upload this as a separate file labeled 'Revised Manuscript with Track Changes'.
- An unmarked version of your revised paper without tracked changes. You should upload this as a separate file labeled 'Manuscript'.

If you would like to make changes to your financial disclosure, please include your updated statement in your cover letter. Guidelines for resubmitting your figure files are available below the reviewer comments at the end of this letter.

If applicable, we recommend that you deposit your laboratory protocols in protocols.io to enhance the reproducibility of your results. Protocols.io assigns your protocol its own identifier (DOI) so that it can be cited independently in the future. For instructions see: https://journals.plos.org/plosone/s/submission-guidelines#loc-laboratory-protocols. Additionally, PLOS ONE offers an option for publishing peer-reviewed Lab Protocol articles, which describe protocols hosted on protocols.io. Read more information on sharing protocols at https://plos.org/protocols?utm_medium=editorial-email&utm_source=authorletters&utm_campaign=protocols.

We look forward to receiving your revised manuscript.

Kind regards,

Wajid Ali

Academic Editor

PLOS One

Journal Requirements:

When submitting your revision, we need you to address these additional requirements.

1. Please ensure that your manuscript meets PLOS ONE's style requirements, including those for file naming. The PLOS ONE style templates can be found at

https://journals.plos.org/plosone/s/file?id=wjVg/PLOSOne_formatting_sample_main_body.pdf and

https://journals.plos.org/plosone/s/file?id=ba62/PLOSOne_formatting_sample_title_authors_affiliations.pdf

2. In your Methods section, please provide additional information regarding the permits you obtained for the work. Please ensure you have included the full name of the authority that approved the field site access and, if no permits were required, a brief statement explaining why.

3. We note that the grant information you provided in the ‘Funding Information’ and ‘Financial Disclosure’ sections do not match.

When you resubmit, please ensure that you provide the correct grant numbers for the awards you received for your study in the ‘Funding Information’ section.

4. Thank you for stating the following financial disclosure: [The research work was financially supported by the National Natural Science Foundation of China (41761097).].

Please state what role the funders took in the study.  If the funders had no role, please state: ""The funders had no role in study design, data collection and analysis, decision to publish, or preparation of the manuscript."

If this statement is not correct you must amend it as needed.

Please include this amended Role of Funder statement in your cover letter; we will change the online submission form on your behalf.

5. PLOS requires an ORCID iD for the corresponding author in Editorial Manager on papers submitted after December 6th, 2016. Please ensure that you have an ORCID iD and that it is validated in Editorial Manager. To do this, go to ‘Update my Information’ (in the upper left-hand corner of the main menu), and click on the Fetch/Validate link next to the ORCID field. This will take you to the ORCID site and allow you to create a new iD or authenticate a pre-existing iD in Editorial Manager.

6. Please include captions for your Supporting Information files at the end of your manuscript, and update any in-text citations to match accordingly. Please see our Supporting Information guidelines for more information: http://journals.plos.org/plosone/s/supporting-information.

7. If the reviewer comments include a recommendation to cite specific previously published works, please review and evaluate these publications to determine whether they are relevant and should be cited. There is no requirement to cite these works unless the editor has indicated otherwise.

Additional Editor Comments:

The authors are advised to carefully incorpritae the reviewer suggestions and improve teh paper accrodingly.

[Note: HTML markup is below. Please do not edit.]

Reviewers' comments:

Reviewer's Responses to Questions

**Comments to the Author**

1. Is the manuscript technically sound, and do the data support the conclusions?

The manuscript must describe a technically sound piece of scientific research with data that supports the conclusions. Experiments must have been conducted rigorously, with appropriate controls, replication, and sample sizes. The conclusions must be drawn appropriately based on the data presented.

Reviewer #1: Partly

Reviewer #2: No

Reviewer #3: No

Reviewer #4: Yes

**********

2. Has the statistical analysis been performed appropriately and rigorously?

Reviewer #1: Yes

Reviewer #2: No

Reviewer #3: No

Reviewer #4: Yes

**********

3. Have the authors made all data underlying the findings in their manuscript fully available?

The PLOS Data policy requires authors to make all data underlying the findings described in their manuscript fully available without restriction, with rare exception (please refer to the Data Availability Statement in the manuscript PDF file). The data should be provided as part of the manuscript or its supporting information, or deposited to a public repository. For example, in addition to summary statistics, the data points behind means, medians and variance measures should be available. If there are restrictions on publicly sharing data—e.g. participant privacy or use of data from a third party—those must be specified.

Reviewer #1: Yes

Reviewer #2: Yes

Reviewer #3: Yes

Reviewer #4: Yes

**********

4. Is the manuscript presented in an intelligible fashion and written in standard English?

PLOS ONE does not copyedit accepted manuscripts, so the language in submitted articles must be clear, correct, and unambiguous. Any typographical or grammatical errors should be corrected at revision, so please note any specific errors here.

Reviewer #1: Yes

Reviewer #2: Yes

Reviewer #3: No

Reviewer #4: Yes

**********

5. Review Comments to the Author

Please use the space provided to explain your answers to the questions above. You may also include additional comments for the author, including concerns about dual publication, research ethics, or publication ethics. (Please upload your review as an attachment if it exceeds 20,000 characters)

Reviewer #1: The manuscript is addressing an iportant problem particulary in Asia with reference to arsenic contamination. However,

I would like to suggest some changes

Figure captions need to be explanatory like P1, P2, and P3 which pH ranges each one is referrirng same is the case wih other treatments. pH 9.5 although hindred Arsenic adsorption on the soil surface. What is the impact of this soil pH on plant growth.

Figure2 Captions need to be more clearly explained. Figure 2 (A-C) No depth point has been mentioned in the figure panel or in the caption.How significant was the difference at which time point incase pf P3 . Figure2 (D-F) Concentration of sal soution used for anaysis not mentioned,. Again the depth should be mentioned in the figure panel/caption.

Reviewer #2: Review of the manuscript entitled “The migration of arsenic in the arid farmland soil under arsenic groundwater irrigation in Xinjiang”, which presents results on the physical and chemical parameters of soil under changing scenarios of evaporation, depth, pH, and salinity.

The manuscript presents several aspects that still require improvement before proceeding to a formal peer-review process.

Major revisions

Objective

The objective of the study is not clearly stated. The objective and title must be described in accordance with the methodology employed and in agreement with the experimental results. This is particularly important because the study is based on controlled experimental conditions rather than direct field application; therefore, the innovative contribution of the present work should be explicitly stated. Furthermore, the abstract mentions that the study area is a reclamation area; however, this is not subsequently reflected in the objective proposed in the Introduction section. Additionally, this creates confusion in the Study Area section (Methodology).

Introduction

Although the Introduction mentions the arsenic situation in the area of interest, it should also describe the different methodologies available for assessing arsenic mobilization, as well as the physical, chemical, and biological dynamics that must be considered in this type of controlled experiment. This is relevant because the study focuses on evaluating the mobilization of this metalloid within a simulated environment.

Methodology

In the Methodology section, several aspects require improvement in accordance with the stated objective of the study.

1. Study site. In this subsection, some results from soil characterization are reported. However, this section should be written in a way that clearly explains that this area corresponds to the source from which soil samples were collected. This can be addressed in different ways:

a) This subsection could be removed, integrating the description of the soil sampling location into the experimental design (which is already briefly described).

b) If this subsection is retained, the area should be described in general terms, including physical aspects such as location, hydrological, climatic, geological/edaphological characteristics, and socioeconomic activities, without repeating information already presented in the Introduction.

c) Are the values of the physicochemical variables for both groundwater and cultivated soil characterization own analyses or obtained from literature? If they are own results, they should be reported in the Results section; if they are taken from previous studies, the corresponding bibliographic references must be provided.

2. Experimental design. The experimental design is not clearly described as currently presented. This subsection should comprehensively detail all procedures performed: sampling sites (number of soil and water sampling sites, and whether they were statistically determined), depth and area (sampling methodology), number of samples per site (quality control, whether composite samples were prepared), soil sample characterization procedures, number of water samples characterized, and whether the water used in the experiment was groundwater or a prepared solution. The basis for selecting pH and salinity values in the water should be clearly described. The meaning of CK, E, and P in Table 2 must be explained. The number of cylindrical pots used per treatment type (TDS, pH, and salinity) should be specified, as well as the rationale for selecting surface and indoor temperatures and the chosen TDS, pH, and salinity values. The criteria for using the membrane arrangement shown in Figure 1 should also be justified. All analyzed variables must be listed, as the Results section reports Eh measurements that are not mentioned in this section.

3. A specific subsection describing the measurement methodologies should be included, covering elemental and physical determinations, as well as a detailed description of the SEM-EDX methodology (morphological and semi-quantitative analysis).

4. Data analysis. The statistical analyses used should be clearly specified and justified. Regarding the kriging evaluation applied to the membrane, it should be clarified whether arsenic concentration was assessed vertically, according to the arrangement shown in Figure 1. The characteristics and parameters of the kriging method applied must be described.

Results and discussion.

Because the experimental design is not clearly defined, the results lack a general framework that explains what is being analyzed to address the stated objective. The analysis of treatments is presented separately; however, the relationships among treatments and their connection with the results obtained from the membrane are not clearly established, and the analysis of the membrane itself is not well understood. To strengthen the manuscript, figures showing morphology and a table with qualitative elemental analysis derived from SEM-EDX should be included.

Based on all the above, I consider that the authors should substantially rewrite the manuscript to improve clarity, strengthen the technical and scientific analysis (supported by statistical analyses within and between treatments), and provide a deeper discussion contrasted with other methodological simulation approaches. This will help clarify the scientific contribution and innovative value of the present study.

Reviewer #3: While the topic of arsenic migration in arid agricultural soils is relevant, the manuscript suffers from fundamental scientific and methodological weaknesses that rule out publication in its current form. The experimental design is overly simplified and does not realistically represent arsenic geochemical behavior in soils, particularly with respect to redox control, mineralogical interactions, and arsenic speciation. The central reliance on ion-exchange membranes combined with SEM-EDS provides only semi-quantitative, unvalidated evidence, yet the results are interpreted as definitive spatial distributions. Data analysis is largely descriptive, lacks proper statistical or mechanistic treatment, and does not advance process-level understanding beyond well-established concepts. Given these substantive limitations, the study does not meet the scientific approach expected for a PLOS ONE research article and would require fundamental redesign rather than revision.

Reviewer #4: the write up is ok,

1. abstract should be revised, objective clearly stated and also methodology the conclusion should be based on the results.

sentences are too long eg. line 13 to 16,

introduction

add current references, some references are too old

strengthen the problem statement

methodology

add map of study area

data analysis - indicate the version of excel

add method validation/ control for the instrument etc

Discussion should be strengthened

**********

6. PLOS authors have the option to publish the peer review history of their article (what does this mean?). If published, this will include your full peer review and any attached files.

If you choose “no”, your identity will remain anonymous but your review may still be made public.

**Do you want your identity to be public for this peer review?**  For information about this choice, including consent withdrawal, please see our Privacy Policy.

Reviewer #1: No

Reviewer #2: No

Reviewer #3: No

Reviewer #4: **Yes:**  David Azanu

**********

[NOTE: If reviewer comments were submitted as an attachment file, they will be attached to this email and accessible via the submission site. Please log into your account, locate the manuscript record, and check for the action link "View Attachments". If this link does not appear, there are no attachment files.]

To ensure your figures meet our technical requirements, please review our figure guidelines: https://journals.plos.org/plosone/s/figures

You may also use PLOS’s free figure tool, NAAS, to help you prepare publication quality figures: https://journals.plos.org/plosone/s/figures#loc-tools-for-figure-preparation.

NAAS will assess whether your figures meet our technical requirements by comparing each figure against our figure specifications.

# Author comment: Author response to Decision Letter 1

**DOI:** 10.1371/journal.pone.0351925.r002

16 Mar 2026

Dear reviewers:

Thankyou for your meticulous and patient work on our article, and for providing us with very useful review comments. We have carefully revised the article based on the review comments. The following is our response to the review comments.

------------------------------

Response to Reviewer #1

Reviewer Comments 1: Figure captions need to be explanatory like P1，P2, and P3 which pH ranges each one is referring same is the case with other treatments.

Response : Thank you for this valuable suggestion. We have revised the captions for all figures to clearly indicate the specific conditions for each treatment. In Fig 1, we have added a note explaining that P1, P2 and P3 correspond to pH 7.5, 8.5 and 9.5, respectively, and that T1, T2 and T3 correspond to TDS 0.5, 1.5 and 4.5 g·L-1, E1, E2 correspond to indoor and outdoor treatment, respectively. The revised can be found in the document titled Figures in red.

Reviewer Comments 2: pH 9.5 although hindered Arsenic adsorption on the soil surface. What is the impact of this soil pH on plant growth?

Response : Dear reviewer, thank you for your raising on this important point. In the revised Discussion section, we have added a paragraph addressing the impact of high pH on plant growth. Specifically, we discuss how high pH environments can disrupt root cell membrane stability and function, altering water and nutrient uptake, and how this, combined with elevated As bioavaliability, may exacerbate the inhibitory effects of As on crop growth. This addition can be found on Page 18, Lines 360-368.

Reviewer Comments 3: Figure 2 Captions need to be more clearly explained. Figure 2 (A-C) No depth point has been mentioned in the figure panel or in the caption. How significant was the difference at which time point in case of P3. Figure 2 (D-F) Concentration of salt solution used for analysis not mentioned. Again the depth should be mentioned in the figure panel/caption.

Response : Thank you for your suggestion. we have revised the figure captions accordingly. In response to Reviewer 4’s comments on adding a map, the original Fig 2 has changed to Fig 3. In the caption for Fig 3, we now explicitly state that the data points at depths of 5 cm, 15 cm and 25 cm are shown from left to right. For the P3 treatment, we have clarified that the As content was significantly higher than P1 and P2 throughout the entire experimental period, as stated on Page 12, Lines 235-238. The salt concentrations for treatments T1, T2 and T3 (0.5, 1.5 and 4.5 g·L-1) are now clearly indicated in the figure captions and Page 8, Table 1.

------------------------------

Response to Reviewer #2

Reviewer Comments 1 (Objective): The objective of the study is not clearly stated. The objective and title must be described in accordance with the methodology employed and in agreement with the experimental results. This is particularly important because the study is based on controlled experimental conditions rather than direct field application; therefore, the innovative contribution of the present work should be explicitly stated. Furthermore, the abstract mentions that the study area is a reclamation area; however, this is not subsequently reflected in the objective proposed in the Introduction section. Additionally, this creates confusion in the Study Area section (Methodology).

Response : Thank you for this important observation. We have changed the title to Simulating arsenic migration in arid farmland soils under high-arsenic groundwater irrigation in Xinjiang to highlight the simulation of the experiment and revised the objective statement in the Introduction to clearly reflect the controlled experimental nature of the study and to highlight the innovative contribution of our methodology. The revised objective now reads: "Based on this, a simulated irrigation controlled experiment was conducted to investigate the migration characteristics of As(V) in soils under different evaporation intensities, pH values and TDS conditions, using cotton field soils from the Kuitun River Basin in Xinjiang as the research object." This can be found on Page 5-6, Lines 103-106. We have also ensured consistency between the abstract and the Introduction by specifying "Kuitun River Basin" throughout the manuscript.

Reviewer Comment 2 (Introduction): Although the Introduction mentions the arsenic situation in the area of interest, it should also describe the different methodologies available for assessing arsenic mobilization, as well as the physical, chemical, and biological dynamics that must be considered in this type of controlled experiment. This is relevant because the study focuses on evaluating the mobilization of this metalloid within a simulated environment.

Response: We agree with the reviewer that a comprehensive methodological background is essential. In the revised Introduction, we have added a paragraph (Page 4, Lines 67-79) that reviews various methods for studying As migration, including field trials, column leaching experiments, and the innovative ion exchange membrane combined with SEM-EDS technique. We have also added a paragraph (Page 5, Lines 90-102) discussing the physical, chemical, and biological factors that influence As behavior in controlled experiments, and explaining why we focused on evaporation, pH, and TDS as key variables in this study.

Reviewer Comment 3 (Study site): In this subsection, some results from soil characterization are reported. However, this section should be written in a way that clearly explains that this area corresponds to the source from which soil samples were collected. This can be addressed in different ways: a) This subsection could be removed, integrating the description of the soil sampling location into the experimental design. b) If this subsection is retained, the area should be described in general terms, including physical aspects such as location, hydrological, climatic, geological/edaphological characteristics, and socioeconomic activities, without repeating information already presented in the Introduction. c) Are the values of the physicochemical variables for both groundwater and cultivated soil characterization own analyses or obtained from literature? If they are own results, they should be reported in the Results section; if they are taken from previous studies, the corresponding bibliographic references must be provided.

Response: Thank you for your three suggestions. Explaining the source of the samples can indeed facilitate readers’ understanding of our research, which is very important to us. We choose to combine suggestions a) and c) for modification. We have revised Section 2.1 to focus on general geographical and environmental descriptions of the Kuitun River Basin. We have clarified that the soil samples were collected from this area and that the groundwater quality data were obtained from our research group's previous study (Deng et al. 2021). The soil characterization results have been moved to the Results section (Section 3.1) as suggested. The revised text can be found on Page 6-7, Lines 122-129 and Page 11-12, Lines 223-236.

Reviewer Comment 4 (Experimental design): The experimental design is not clearly described as currently presented. This subsection should comprehensively detail all procedures performed: sampling sites (number of soil and water sampling sites, and whether they were statistically determined), depth and area (sampling methodology), number of samples per site (quality control, whether composite samples were prepared), soil sample characterization procedures, number of water samples characterized, and whether the water used in the experiment was groundwater or a prepared solution. The basis for selecting pH and salinity values in the water should be clearly described. The meaning of CK, E, and P in Table 2 must be explained. The number of cylindrical pots used per treatment type (TDS, pH, and salinity) should be specified, as well as the rationale for selecting surface and indoor temperatures and the chosen TDS, pH, and salinity values. The criteria for using the membrane arrangement shown in Figure 1 should also be justified. All analyzed variables must be listed, as the Results section reports Eh measurements that are not mentioned in this section.

Response: Thank you for patiently pointing out the shortcomings in our experimental design description. We have substantially expanded Section 2.2 to include all requested details:

Sampling method: Five-point sampling method, composite sample preparation (Page 6, Lines 124-126)

Water source: Laboratory-prepared solution using Na₃AsO₄·12H₂O (Page 7, Line 144-145)

pH and TDS selection: Based on natural variation range of groundwater in the Kuitun River Basin (Page 7, Lines 140-143)

Treatment abbreviations: Explained in Table 1 note (Page 8, Lines 152-154)

Number of replicates: Each treatment performed in triplicate (Page 7, Line 143-144)

Temperature rationale: Indoor 22–25°C (constant temperature to minimize evaporation), outdoor 31–36°C (typical summer daytime temperatures) (Page 7, Lines 145-148)

Membrane arrangement: Justified on Page 8-9, Lines 159-166 and supplemented with references on the use of similar ion membrane installation methods.

All analyzed variables: Eh, pH, total As in soil solution, and As distribution on membranes are now explicitly listed (Page 9-10, Lines 178-203)

Reviewer Comment 5: A specific subsection describing the measurement methodologies should be included, covering elemental and physical determinations, as well as a detailed description of the SEM-EDX methodology (morphological and semi-quantitative analysis).

Response: Thank you for your advice. We have added Section 2.3 "Measurement methodologies" (Page 9-10, Lines 178-203) that includes detailed descriptions of all analytical methods, including SEM-EDS operating conditions (accelerating voltage 10 kV, working distance 10 mm, acquisition time ~2 min per point).

Reviewer Comment 6 (Data analysis): The statistical analyses used should be clearly specified and justified. Regarding the kriging evaluation applied to the membrane, it should be clarified whether arsenic concentration was assessed vertically, according to the arrangement shown in Figure 1. The characteristics and parameters of the kriging method applied must be described.

Response: Dear reviewer, we have revised Section 2.4 to specify that one-way ANOVA was used to test significance (P < 0.05). For the kriging analysis, we clarified that vertical As migration was assessed by analyzing membrane sections at different depths, and horizontal migration by comparing sections at the same depth but different radial positions, and supplemented the content of Fig 2 to facilitate readers’ understanding of the method for determining arsenic content on ion exchange membranes. Default kriging parameters in Surfer 13.0 were used, as the focus was on visualizing relative distribution patterns. These details can be found on Page 10-11, Lines 204-213.

Reviewer Comment 7 (Results and discussion): Because the experimental design is not clearly defined, the results lack a general framework that explains what is being analyzed to address the stated objective. The analysis of treatments is presented separately; however, the relationships among treatments and their connection with the results obtained from the membrane are not clearly established, and the analysis of the membrane itself is not well understood. To strengthen the manuscript, figures showing morphology and a table with qualitative elemental analysis derived from SEM-EDX should be included.

Response: We have restructured the Results section to improve clarity and establish clear connections between the soil solution data and membrane results. Section 3.2 presents the soil solution data for pH, salt, and evaporation conditions, while Section 3.3 presents the membrane results, with each subsection explicitly referencing the corresponding findings from Section 3.2.(Page 14-16, Lines 279-320).

------------------------------

Response to Reviewer #3

Reviewer Comments: While the topic of arsenic migration in arid agricultural soils is relevant, the manuscript suffers from fundamental scientific and methodological weaknesses that rule out publication in its current form. The experimental design is overly simplified and does not realistically represent arsenic geochemical behavior in soils, particularly with respect to redox control, mineralogical interactions, and arsenic speciation. The central reliance on ion-exchange membranes combined with SEM-EDS provides only semi-quantitative, unvalidated evidence, yet the results are interpreted as definitive spatial distributions. Data analysis is largely descriptive, lacks proper statistical or mechanistic treatment, and does not advance process-level understanding beyond well-established concepts.

Response:We thank the reviewer for their critical assessment, which has helped us significantly improve the manuscript. We have addressed these concerns as follows:

1.Experimental design complexity: In the revised Introduction (Page 5, Lines 90-102), we now explicitly acknowledge the complexity of As migration in natural systems, including redox control, mineralogical interactions, and biological factors. We explain that our study focuses on three key factors (evaporation, pH, and TDS) as a controlled approach to isolate their effects, while recognizing that these interactions occur within a more complex natural system.

2.Methodology validation: We have clarified that the ion exchange membrane combined with SEM-EDS provides spatial distribution information that corroborates the quantitative measurements of As in soil solutions, rather than replacing them. This distinction is now clearly stated in the Abstract (Page 2, Lines 14-18) and in Introduction (Page 6, Lines 106-110). The two methods are presented as complementary rather than definitive alone.

3.Statistical and mechanistic analysis: We have strengthened the data analysis by:

·Adding one-way ANOVA to test significance of differences among treatments (Section 2.4, Page 10-11, Lines 206-208);

·Incorporating mechanistic explanations in the Discussion, including mineral adsorption mechanisms (Fe/Mn oxides forming inner-sphere complexes), pH effects (OH- competition, electrostatic adsorption), salinity effects (double layer compression, ion exchange), and evaporation effects (hydraulic gradients). These can be found throughout Section 4 (Page 16-18, Lines 328-333, 357-359, 374-378, 384-387).

4.Process-level understanding: The revised Discussion now advances beyond well-established concepts by:

·Revealing the "hemispherical" high-As zone near the drip emitter, which traditional methods cannot capture

·Demonstrating that high pH and salinity not only increase As release but also change its migration direction from horizontal diffusion to vertical leaching

·Providing spatial evidence that distinguishes between desorption and migration processes

We believe these revisions have substantially strengthened the scientific rigor of our study and clarified its contribution to understanding As migration under controlled irrigation conditions.

------------------------------

Response to Reviewer #4

Reviewer Comments 1: Abstract should be revised, objective clearly stated and also methodology the conclusion should be based on the results. Sentences are too long (e.g., line 13 to 16).

Response: Thank you for your suggestions. We have revised the Abstract to ensure the objective is clearly stated, the methodology is concise, and the conclusions are directly based on the results. Long sentences have been split into shorter, clearer statements. The revised Abstract can be found on Page 2, Lines 9-25.

Reviewer Comment 2: Introduction - add current references, some references are too old. Strengthen the problem statement.

Response: Thank you for your advice, adding recent references can facilitate readers to have a clearer understanding of the current research status. We have added several recent references (2020-2025) throughout the Introduction, including Asamoah-Ntow et al. (2025), Tran et al. (2023), Viana et al. (2022), and Morosini et al. (2023). The problem statement has been strengthened by adding a global perspective: "For instance

Attachment Submitted filename: Response to Reviewers.docx

# Peer review: Decision Letter 1

**DOI:** 10.1371/journal.pone.0351925.r003

**Authors:** Wajid Ali, Wajid Ali

3 Jun 2026

<p>Simulation arsenic migration in arid farmland soils under high-arsenic groundwater irrigation in Xinjiang

PONE-D-25-46672R1

Dear Dr. Luo,

We’re pleased to inform you that your manuscript has been judged scientifically suitable for publication and will be formally accepted for publication once it meets all outstanding technical requirements.

Within one week, you’ll receive an e-mail detailing the required amendments. When these have been addressed, you’ll receive a formal acceptance letter and your manuscript will be scheduled for publication.

An invoice will be generated when your article is formally accepted. Please note, if your institution has a publishing partnership with PLOS and your article meets the relevant criteria, all or part of your publication costs will be covered. Please make sure your user information is up-to-date by logging into Editorial Manager at Editorial Manager® and clicking the ‘Update My Information' link at the top of the page. For questions related to billing, please contact billing support.

If your institution or institutions have a press office, please notify them about your upcoming paper to help maximize its impact. If they’ll be preparing press materials, please inform our press team as soon as possible -- no later than 48 hours after receiving the formal acceptance. Your manuscript will remain under strict press embargo until 2 pm Eastern Time on the date of publication. For more information, please contact onepress@plos.org.

Kind regards,

Wajid Ali

Academic Editor

PLOS One

Additional Editor Comments (optional):

Reviewers' comments:

Reviewer's Responses to Questions

**Comments to the Author**

1. If the authors have adequately addressed your comments raised in a previous round of review and you feel that this manuscript is now acceptable for publication, you may indicate that here to bypass the “Comments to the Author” section, enter your conflict of interest statement in the “Confidential to Editor” section, and submit your "Accept" recommendation.

Reviewer #1: All comments have been addressed

Reviewer #3: (No Response)

**********

2. Is the manuscript technically sound, and do the data support the conclusions?

The manuscript must describe a technically sound piece of scientific research with data that supports the conclusions. Experiments must have been conducted rigorously, with appropriate controls, replication, and sample sizes. The conclusions must be drawn appropriately based on the data presented.

Reviewer #1: Yes

Reviewer #3: Partly

**********

3. Has the statistical analysis been performed appropriately and rigorously?

Reviewer #1: No

Reviewer #3: N/A

**********

4. Have the authors made all data underlying the findings in their manuscript fully available?

The PLOS Data policy requires authors to make all data underlying the findings described in their manuscript fully available without restriction, with rare exception (please refer to the Data Availability Statement in the manuscript PDF file). The data should be provided as part of the manuscript or its supporting information, or deposited to a public repository. For example, in addition to summary statistics, the data points behind means, medians and variance measures should be available. If there are restrictions on publicly sharing data—e.g. participant privacy or use of data from a third party—those must be specified.

Reviewer #1: Yes

Reviewer #3: No

**********

5. Is the manuscript presented in an intelligible fashion and written in standard English?

PLOS ONE does not copyedit accepted manuscripts, so the language in submitted articles must be clear, correct, and unambiguous. Any typographical or grammatical errors should be corrected at revision, so please note any specific errors here.

Reviewer #1: Yes

Reviewer #3: Yes

**********

6. Review Comments to the Author

Please use the space provided to explain your answers to the questions above. You may also include additional comments for the author, including concerns about dual publication, research ethics, or publication ethics. (Please upload your review as an attachment if it exceeds 20,000 characters)

Reviewer #1: All the comments have been addressed,No further revisions are required for this manuscript. Thanks alot.

Reviewer #3: Although the revised version of this manuscript shows that the main scientific issues presented in the earlier round are addressed in a better manner, the overall scientific issues brought up during the previous round are still not fully addressed. The authors have made some improvements in the framing of the study and in clarifying the role of the ion exchange membrane combined with SEM-EDS but the revisions have predominantly made the study easier to see and interpret descriptively but have not truly offered any real mechanistic progress of arsenic geochemical processes. The experimental design still falls short of adequately addressing redox control, As speciation (As(III)/As(V)) or mineralogical interactions, or reactive transport mechanisms which are essential to understanding arsenic behavior in soils. The recognition of complexity in the Introduction is not a replacement of the fact that it must be incorporated in the experimental or analytic system.

To make the manuscript sufficiently strong in the mechanistic aspect of the study, the authors will have to significantly reinforce this aspect of the work. In particular they should (i) attempt to provide quantitative validation of the ion exchange membrane method (e.g., calibration tests, recovery experiments, correlation with porewater As concentrations, uncertainty analysis), (ii) better attempt to integrate Eh dynamics and Fe/Mn oxide interactions into the analysis with supporting quantitative reasoning based on experimental or literature work, and (iii) temper claims of process-level discovery unless supported by kinetic, sorption, or transport modeling. The hemispherical pattern of distributions and vertical migration patterns with drip irrigation, as they are currently presented, reflect improved visualization of the processes of drip irrigation, but not an evident increase in the mechanisms of As migration. It would be a great improvement to the scientific richness, and to make sure that the conclusions are backed by mechanistic evidence and not by primarily descriptive spatial patterns.

**********

7. PLOS authors have the option to publish the peer review history of their article (what does this mean?). If published, this will include your full peer review and any attached files.

If you choose “no”, your identity will remain anonymous but your review may still be made public.

**Do you want your identity to be public for this peer review?**  For information about this choice, including consent withdrawal, please see our Privacy Policy.

Reviewer #1: No

Reviewer #3: No

**********

# Editor report: Acceptance letter

**DOI:** 10.1371/journal.pone.0351925.r004

**Authors:** Wajid Ali, Wajid Ali

PONE-D-25-46672R1

PLOS One

Dear Dr. Luo,

I'm pleased to inform you that your manuscript has been deemed suitable for publication in PLOS One. Congratulations! Your manuscript is now being handed over to our production team.

At this stage, our production department will prepare your paper for publication. This includes ensuring the following:

* All references, tables, and figures are properly cited

* All relevant supporting information is included in the manuscript submission,

* There are no issues that prevent the paper from being properly typeset

You will receive further instructions from the production team, including instructions on how to review your proof when it is ready. Please keep in mind that we are working through a large volume of accepted articles, so please give us a few days to review your paper and let you know the next and final steps.

Lastly, if your institution or institutions have a press office, please let them know about your upcoming paper now to help maximize its impact. If they'll be preparing press materials, please inform our press team within the next 48 hours. Your manuscript will remain under strict press embargo until 2 pm Eastern Time on the date of publication. For more information, please contact onepress@plos.org.

You will receive an invoice from PLOS for your publication fee after your manuscript has reached the completed accept phase. If you receive an email requesting payment before acceptance or for any other service, this may be a phishing scheme. Learn how to identify phishing emails and protect your accounts at https://explore.plos.org/phishing.

If we can help with anything else, please email us at customercare@plos.org.

Thank you for submitting your work to PLOS ONE and supporting open access.

Kind regards,

PLOS ONE Editorial Office Staff

on behalf of

Dr. Wajid Ali

Academic Editor

PLOS One
